# Qualitative Analysis of Air Freshener Spray

**DOI:** 10.1155/2019/9316707

**Published:** 2019-11-05

**Authors:** Fatima Ibrahim ALshaer, Dalal Fuad ALBaharna, Hafiz Omer Ahmed, Mohammed Ghiyath Anas, Jasem Mohammed ALJassmi

**Affiliations:** Department of Environmental Health, College of Health Sciences, University of Sharjah, Sharjah, UAE

## Abstract

Air fresheners contain various chemicals that may or may not be harmful to human health and the environment. These products are widely used in different settings such as homes, schools, offices, and hospitals with ignorance of their real ingredients and their relative health effects. Thus, this preliminary study was carried out to identify the presence of different compounds in spray air fresheners that were not disclosed on the product's label. Four different brands of spray air fresheners were selected randomly from a local store, in which two were of mid-to-high cost and the remaining two of low cost. The samples were analyzed using gas chromatography/mass spectrometry headspace, in which single components of the samples were identified by the mass spectrometry detector. The results were shown as a chromatogram of several peaks, each representing different compounds. The chemicals found in the samples include; lilial, galaxolide, benzenemethanol, musk ketone, butylated hydroxytoluene, and linalool. These chemicals may cause irritation and other health problems. However, none of them were revealed on the product's label. The study concludes that air fresheners need to be free of any toxic or harmful chemicals and include natural ingredients instead.

## 1. Introduction

Air fresheners are chemical products that have been used in the field of environmental sanitation for decades [1]. These products are used in different settings, including dwellings, hospitals, offices, schools, hotels, restrooms etc. They are available in various forms such as incense, scented candles, oils, disks, aerosol sprays, electric diffusers, and gels. According to Jung et al. [2], air fresheners are indiscriminately used to mask the effects of the deodorizing and fragrant components in indoor environments.

The main purpose of using air fresheners is to get rid of disturbing odours that may result from different activities or processes within an area. They may consist of several ingredients that have the ability to provide a pleasant ambience. Nevertheless, drawbacks may also result due to their excessive usage. They consist of many chemicals that are not revealed on the product label as manufacturers are not required to disclose all ingredients [3]. These chemicals could be allergens, irritants, or even toxic [4]. Steinemann et al. [5] found numerous chemicals in air fresheners, such as acetaldehyde, acetone, benzaldehyde, and limonene that were not listed on the product label.

Numerous studies have been carried out by different researchers on the composition of air fresheners and their relative health effects. For instance, Fleming indicated that some compounds in such products including benzene derivatives, pinene and limonene, aldehydes, phenol, and cresol may pose serious health effects when reacting with other indoor pollutants. Other common chemicals that could be found in air fresheners include VOCs such as benzyl alcohol, toluene, myrcene, phthalates, artificial musks, lilial, and linalool [6, 7].

Air fresheners have been recognized as a primary source of volatile organic compounds throughout buildings from an indoor air-quality perspective. However, air fresheners have been related with adverse effects such as asthma attack, mucosal symptoms, infant illness, breathing difficulties, and migraine headaches from a health perspective [6]. In the previous two national surveys of the US population, breathing difficulties, headaches, and other health problems were reported by 19 percent population when exposed to deodorizers and air fresheners. 10.9 percent population reported health problems from the scent of laundry products vented outdoors [7, 8].

The contribution of a range of risk factors has been assessed by the World Health Organization to the stress of disease and indicated indoor pollution as the 8^th^ most important risk factor and accountable for 2.7 percent of the burden of disease, globally. Every year, indoor air pollution is accountable for the death of 20 individuals [9]. Concentrations of a number of volatile organic compounds are consistently higher indoors as compared to outdoors. Volatile organic compounds are comprised within household products, such as wood preservatives, aerosol sprays, cleansers, disinfectants, paints, paint strippers, moth repellents, air fresheners, hobby supplies, dry-cleaned clothing, stored fuels and automated products, and other solvents [10].

A National Environment Health Action Plan (NEHAP) has been launched by the French government in June 2004, for improving indoor air quality. The Ministries of Health, Labour, and Environment for evaluating health risks related with formaldehyde and other volatile organic ompounds indoors have been mandated by the French Agency for Environmental and Occupational Health Safety (AFSSET). In this regard, the role of AFSSET was to recognize everyday life products comprising or emitting formaldehyde and examine quantity-related human exposure by either direct or indirect sources [11].

Information lacks concerning the gaseous emissions of fragrance products in spite of the extensive indoor exposure and widespread use of fragrances to them [12]. In addition, 95 percent of the chemicals are synthetic compounds in fragrances that are derived from petroleum [13]. Humans have been exposed to specific compounds for assessing the safety of those compounds inhaled when examining the hazard of fragrance compounds [14].

It is important to carry out this study to increase awareness and understanding of their hidden ingredients becasue of the extensive use of air fresheners with the ignorance of their actual contents and their relative effects on humans and the environment. Therefore, the present study aims to identify the presence of different compounds in spray air fresheners that are not disclosed on the product's label.

## 2. Material and Methods

### 2.1. Study Design and Sample Collection

A qualitative, cross-sectional study was conducted to determine the presence of toxic chemicals in the air fresheners. Four different brands of spray air fresheners were selected randomly from a local store, in which two were of mid-to-high cost and the remaining two of low cost.

### 2.2. Sample Analysis

Gas chromatography/mass spectrometry (GC/MS) headspace has been used to analyse the samples obtained from the air fresheners in environmental and analytical chemistry laboratories for the segregation and analysis of readily volatile compounds. The GC 3900/Saturn 2100T GC/MS (ion trap) system was controlled using a Varian GC/MS workstation version 5.52 software. A volume of 1 *μ*L sample was injected into the column, where the separation of the chemical compounds in the air fresheners was performed using an HP VF-5 ms, (30 m × 0.32 mm, 0.25 *μ*m). Helium was used as the carrier gas at a flow rate of 1.0 mL/min, and the injector temperature was at 230°C. The temperature program of the column oven was started at 50°C, was held for 1 min, ramped at 7°C/min to 250°C, and held for 5 mins.

The conditions for the ion trap mass spectrometer were as follows:

- Ionization mode; EI (70 ev)
- Target 5000
- Prescan ionization time 1500 *μ*s
- Scan time 0.66 s
- Scan mode (50–650 M/z)
- Ion trap temperature was at 200°C
- Transfer line temperature 220°C
- Manifold temperature at 45°C.

Finally, the National Institute of Standards and Technology 05 (NIST) mass spectral library was used for the identification of the obtained peaks.  Table 1 shows brief description of each chemicals used in sample freshener.

## 3. Results

The chemicals found after the analysis of the four spray air fresheners include galaxolide, lilial, benzenemethanol, musk ketone, butylated hydroxytoluene (BHT), and linalool. The chromatograms with peaks representing each component found in the samples, along with their boiling points and molecular weights have been illustrated in Figures 1–18.

Table 2 presents the product labels that were labeled and not labeled on the air fresheners. Some chemicals after analysis turned out to be noted as skin allergens or irritants and even chemicals that may interfere with bodily functions. Some other chemicals found were safe to use in fragrance and household products and did not pose any health risk.

## 4. Discussion

The frequent use of air fresheners containing such synthetic chemicals may cause health effects on the long run; although exact concentrations of the chemicals present are not known. The present study has not included any laboratory tests or assessment regarding the potential health effects of air fresheners, as the main emphasis was on the identification of the chemical compounds found in the products. Lilial is found among two of the four samples analyzed that can sometimes act as an allergen and cause contact dermatitis in susceptible individuals [15]. The Campaign for Safe Cosmetics and The Environmental Working Group confirmed that Lilial is found in fragrance products, such as perfumes, colognes, and body sprays [16]. Galaxolide was found in two of the medium-to-high cost air fresheners. This chemical may interfere with hormones and other chemical signals in the body resulting in developmental, reproductive, metabolic, brain, and behavioural problems. Women's Voice for the Earth (WVE) revealed the presence of this chemical in cleaning products, including air fresheners [17].

Benzenemethanol, also known as benzyl alcohol, was found in both the low-cost and medium-to-high cost air fresheners. It is a known irritant when used in cosmetics and causes many problems and abnormalities. The Danish Environmental Protection Agency confirmed the presence of benzyl alcohol in specific fragrance ingredients used in air fresheners and other fragrance liberating products [18]. A previous study undertaken by The Campaign for Safe Cosmetics along with The Environmental Working Group found that BHT and musk ketone were present in fragrance products [19, 20]. Both of these chemicals are associated with estrogenic effects. BHT and musk ketone were found to be present only in the low-cost air fresheners. Finally, linalool is considered to be safe unless found in the oxidized form and causes skin reactions [20]. In the present study, this chemical was found in the low-cost air freshener only. A previous study by Steinemann [6] confirmed that linalool, a terpenoid, is emitted by air fresheners.

Previous studies have used other prevalent volatile organic compounds (VOC) such as acetaldehyde, ethanol, beta-myrcene, acetone, beta-pinene, limonene, and alpha-pinene in at least 40% of the products. Similarly, ethanol, acetaldehyde, alpha pinene, phenoxyethanol, limonene, and ethyl butyrate were the six most prevalent VOC's in building materials detected by GC/MS [21, 22]. These compounds, however, were not discussed and monitored in the present study. Furthermore, in air sampling, VOC's were not detected, which were obtained once daily from one site outside the hospital. Aromatic hydrocarbons such as ethylbenzene, toluene, and xylenes, esters (butyl acetate and ethyl acetate), and aliphatic hydrocarbons (n-hexane) were monitored and detected with TVOC concentrations surpassing the suggested maximum concentration (400 *μ*g/m3) before occupying the building [23]. However, the study missed these chemicals or compounds to be monitored and detected, and thus failed to detect them in air fresheners sprays.

## 5. Conclusion

The present study has identified the presence of different compounds in spray air fresheners that were not disclosed on the product's label. The results depicted common compounds in both low- and high-cost air fresheners. Chemicals found in this study were not revealed on the product label as manufacturers are not required to list all ingredients. These chemicals usually tend to be listed on the product label as “parfum” or “fragrance”. There should be a law that strictly indicates whether the products contain any synthetic chemicals for people to be aware of what they are exposed to, although, manufacturers are not required to reveal all hidden ingredients on the label as stated by the Consumer Product Safety Commission (CPSC). However, manufacturers should be encouraged to start producing air fresheners that are free from any synthetic and toxic chemicals, focusing on the use of natural ingredients instead. People must be aware that air fresheners come with unintended and perhaps invisible risks; therefore, the use of natural practices should be advocated to provide a pleasant ambience. A quantitative analysis of air fresheners is recommended as the present study has only focused on the qualitative analysis. Moreover, the enquiry included solely the emissions of primary VOC of each air freshener without the consideration of secondary pollutants. The limitations of this study were that the version of the GC/MS used was not able to detect highly volatile compounds, so different chemicals other than that detected could also be present in the four air fresheners. Furthermore, terpenes should be detected in future studies as they are found in high abundance. Further studies are needed to guarantee the safety of the product's content and whether present chemicals pose any health risk to humans and the environment.

## Figures and Tables

**Figure 1:**
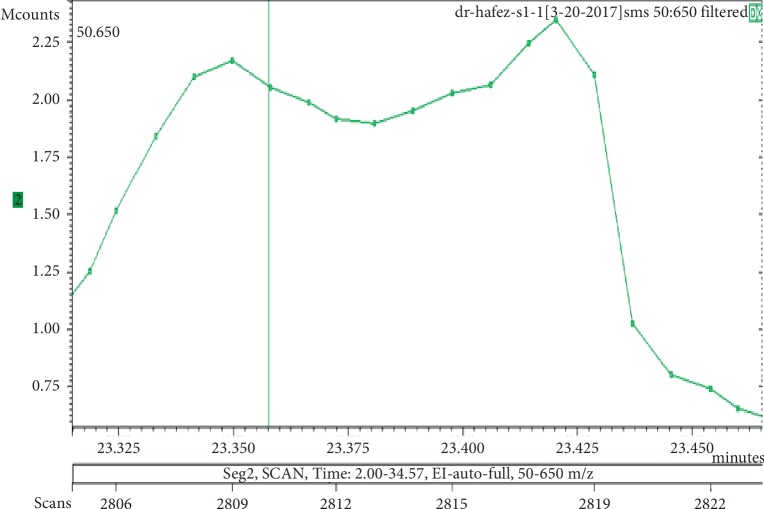
Chromatogram of galaxolide in air freshener sample 1.

**Figure 2:**
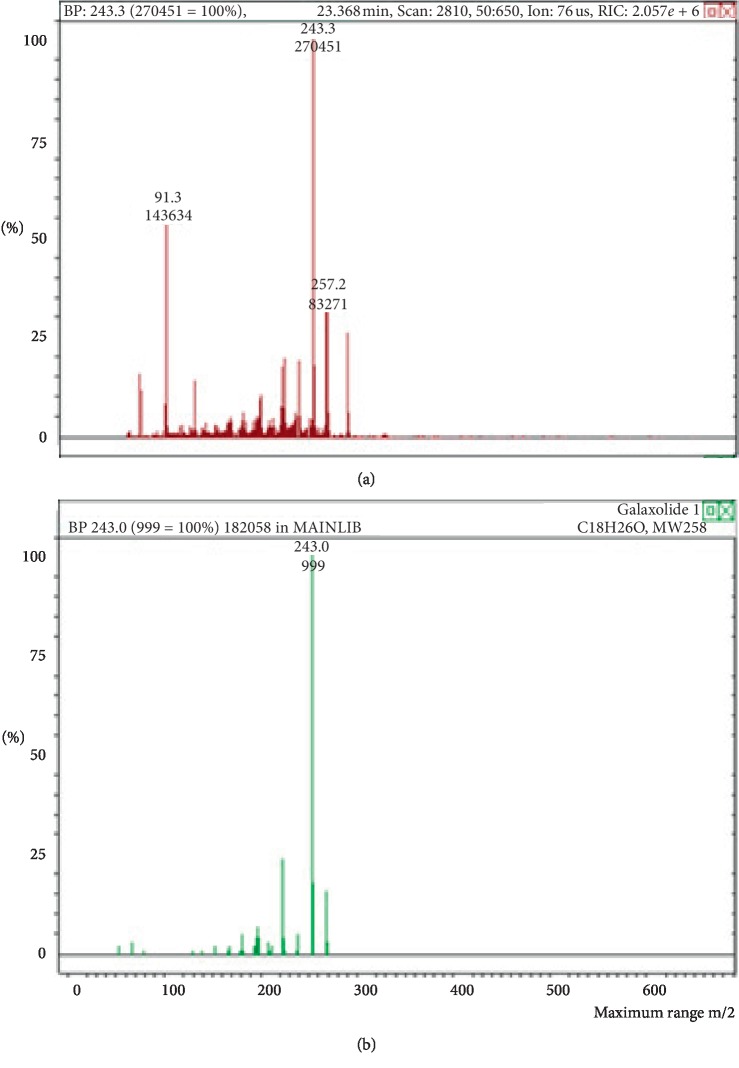
Mass spectrum of galaxolide in air freshener sample 1. (a) Air freshener analysis. (b) Main library of NIST11.

**Figure 3:**
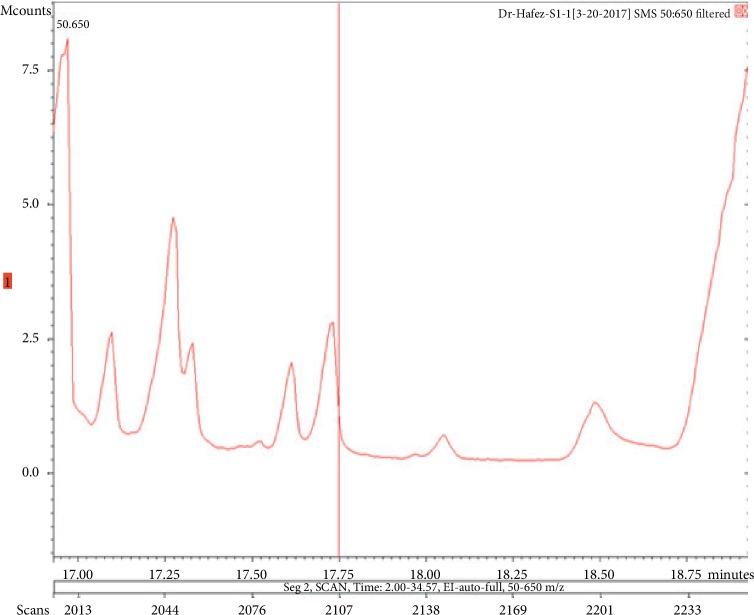
Chromatogram of lilial in air freshener sample 1.

**Figure 4:**
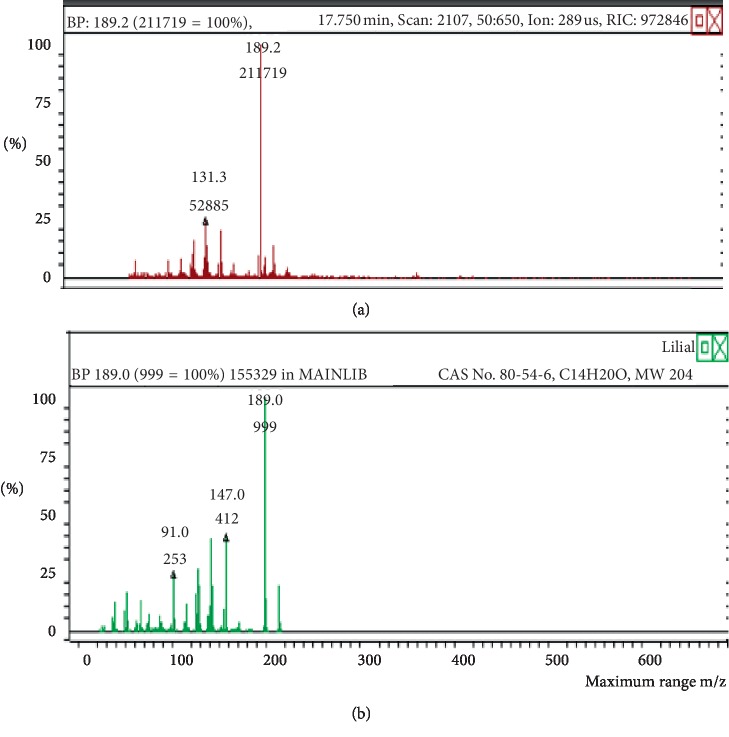
Mass spectrum of lilial in air freshener sample 1. (a) Air freshener analysis. (b) Main library of NIST11.

**Figure 5:**
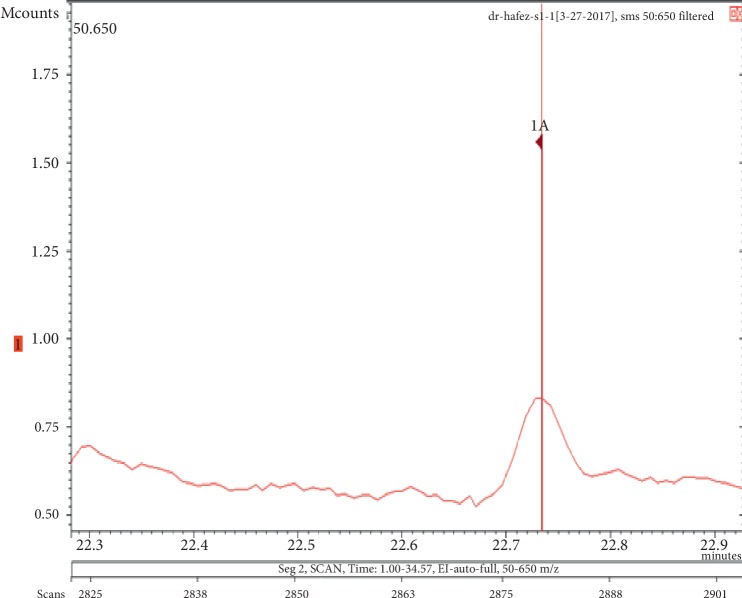
Chromatogram of galaxolide in air freshener sample 2.

**Figure 6:**
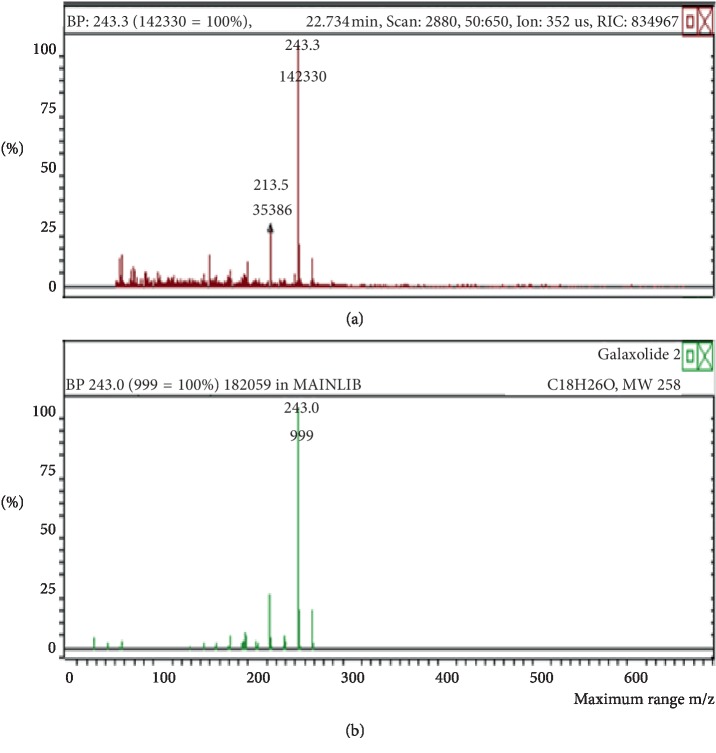
Mass spectrum of galaxolide in air freshener sample 2. (a) Air freshener analysis. (b) Main library of NIST11.

**Figure 7:**
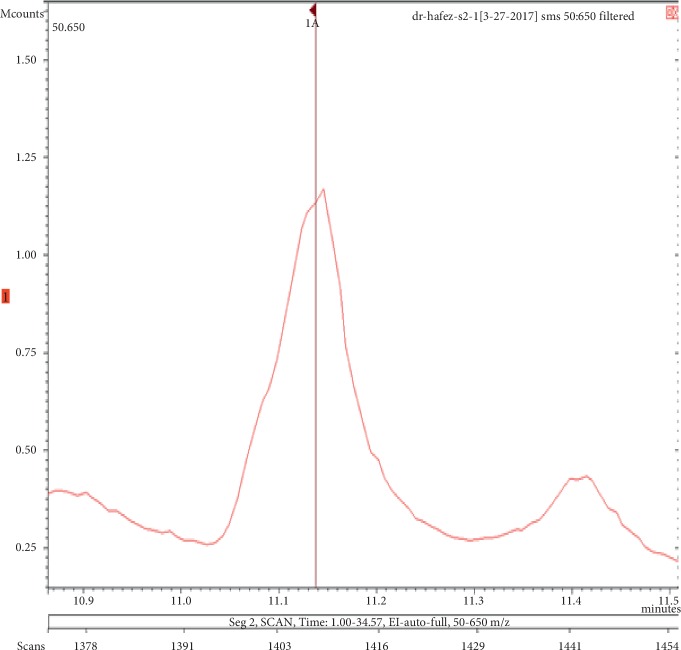
Chromatogram of benzenemethanol in air freshener sample 2.

**Figure 8:**
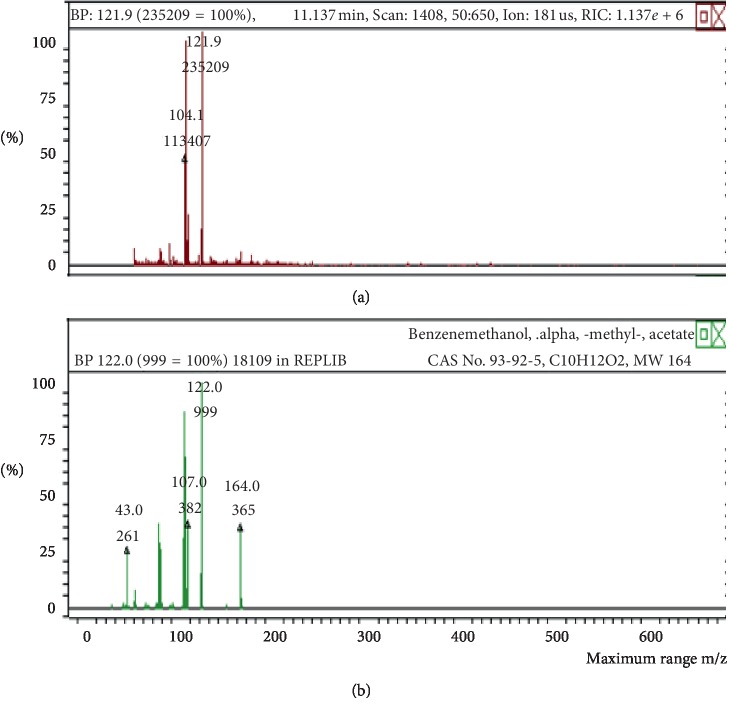
Mass spectrum of benzenemethanol in air freshener sample 2. (a) Air freshener analysis. (b) Main library of NIST11.

**Figure 9:**
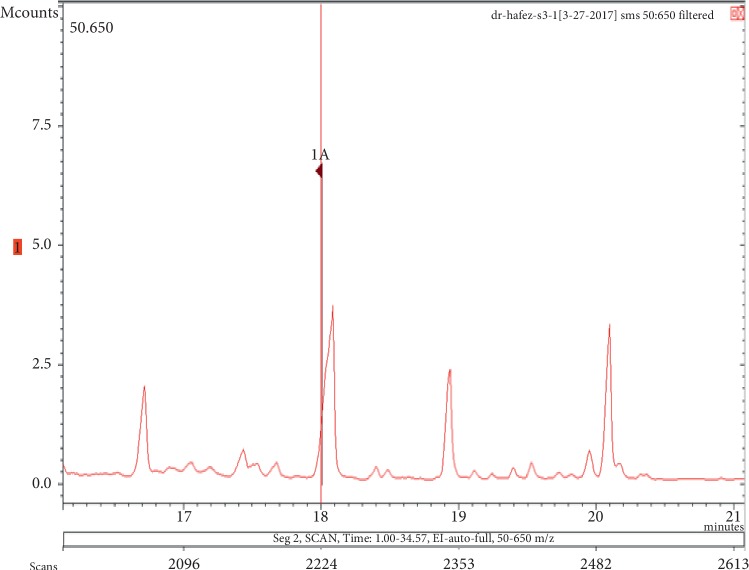
Chromatogram of benzenemethanol in air freshener sample 3.

**Figure 10:**
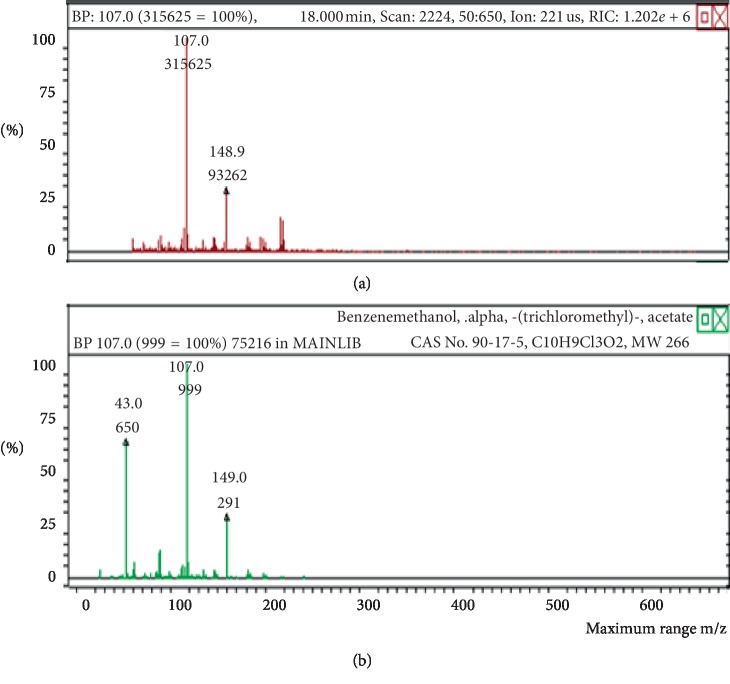
Mass spectrum of benzenemethanol in air freshener sample 3. (a) Air freshener analysis. (b) Main library of NIST11.

**Figure 11:**
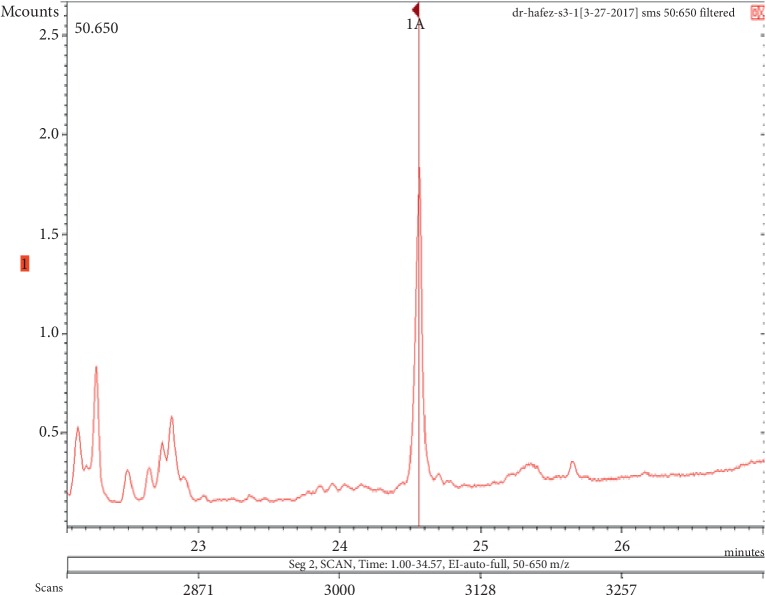
Chromatogram of musk ketone in air freshener sample 3.

**Figure 12:**
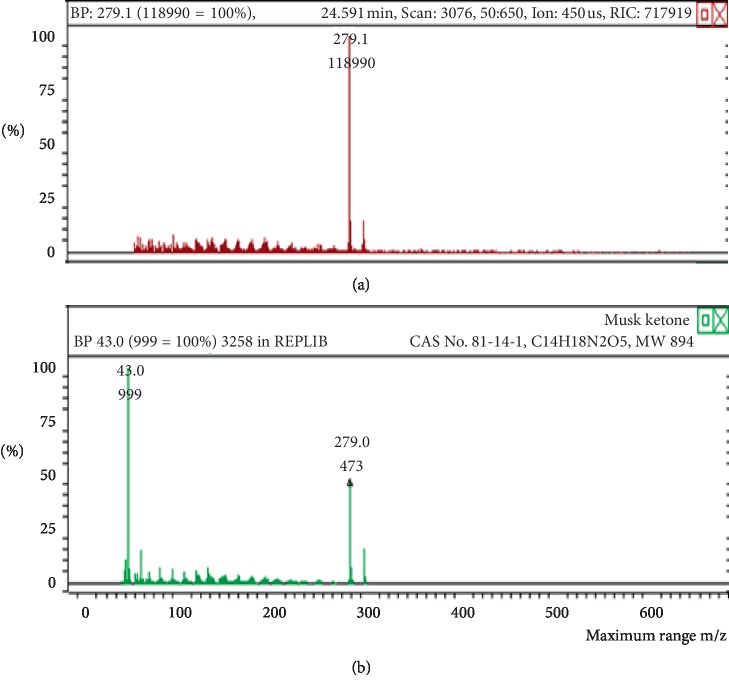
Mass spectrum of musk ketone in air freshener sample 3. (a) Air freshener analysis. (b) Main library of NIST11.

**Figure 13:**
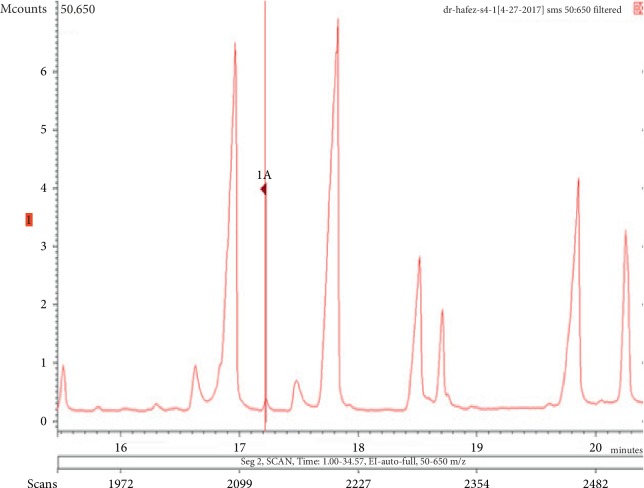
Chromatogram of butylated hydroxytoluene in air freshener sample 4.

**Figure 14:**
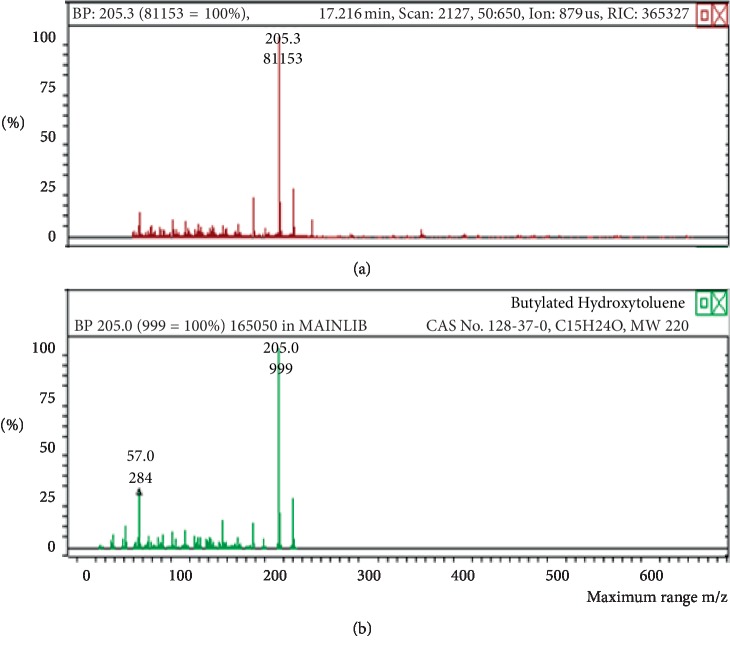
Mass spectrum of butylated hydroxytoluene in air freshener sample 4. (a) Air freshener analysis. (b) Main library of NIST11.

**Figure 15:**
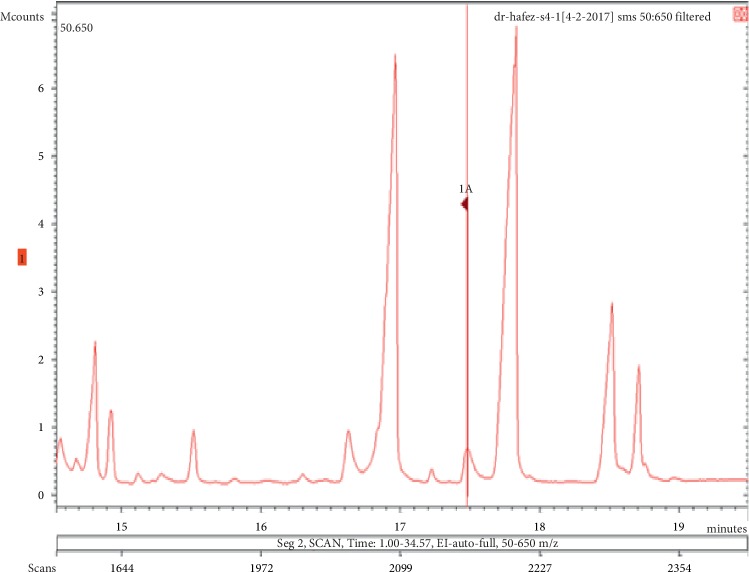
Chromatogram of lilial in air freshener sample 4.

**Figure 16:**
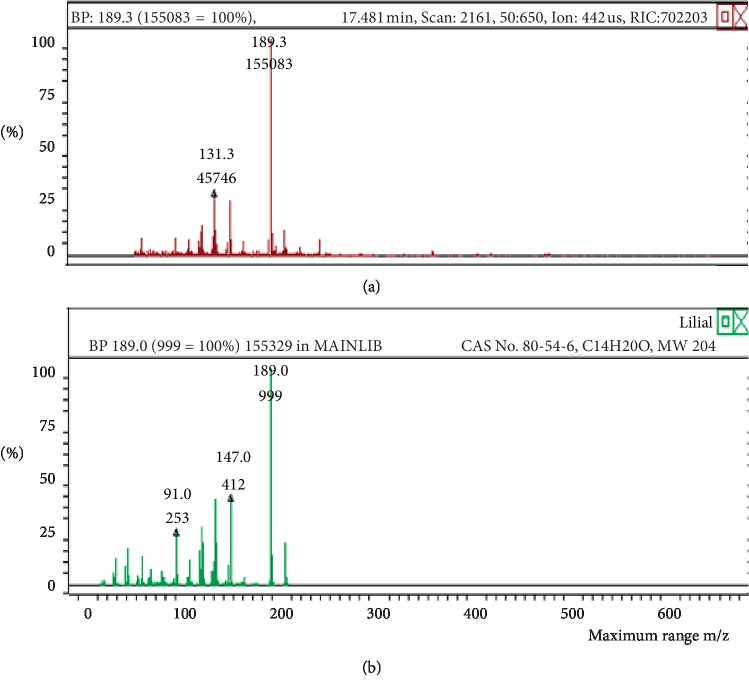
Mass spectrum of lilial in air freshener sample 4. (a) Air freshener analysis. (b) Main library of NIST11.

**Figure 17:**
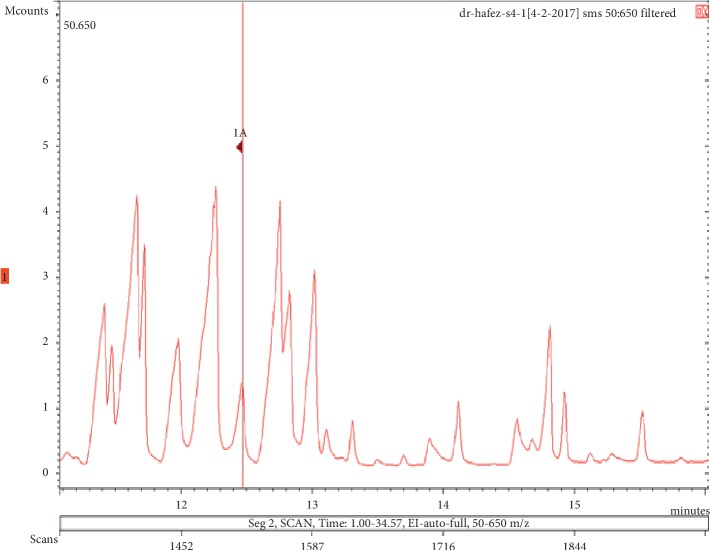
Chromatogram of linalool in air freshener sample 4.

**Figure 18:**
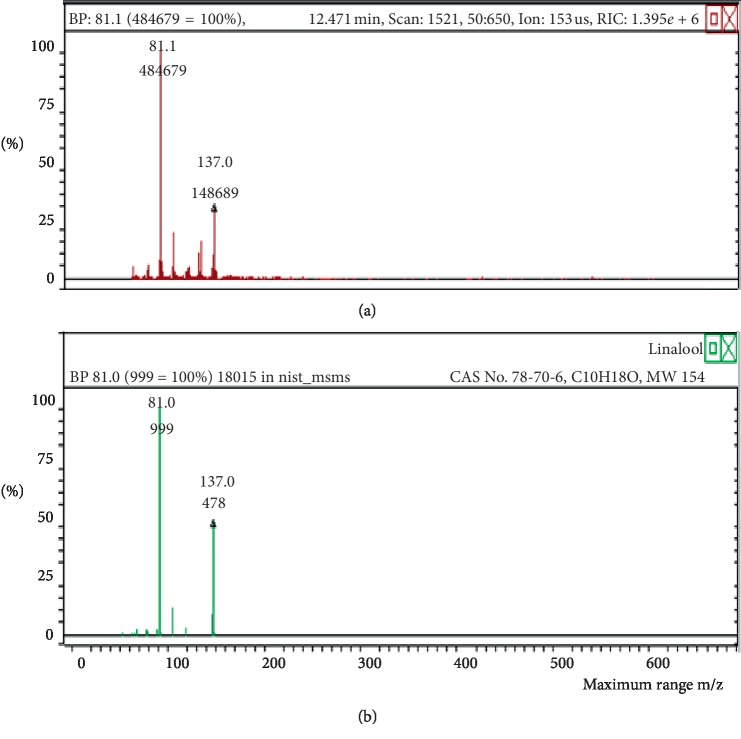
Mass spectrum of linalool in air freshener sample 4. (a) Air freshener analysis. (b) Main library of NIST11.

**Table 1:** Brief description of each chemicals used in the sample freshener.

Sample	Cost	Packaging	Usage	Quantity	CAS
Galaxolide	AED 105	Glass bottle	Aroma/fragrance preparations	30 ml (1 fluid ounce)	1222-05-5
Lilial	AED 274	Glass bottle	Aroma/fragrance preparations	50 ml	80-54-6
Benzenemethanol	AED 124	—	—	16 oz	202-859-9
Musk ketone	AED 76.80	Drum	Perfumery compound	5.25 kgs	—
Butylated hydroxytoluene	AED 12.80	Customized	Perfumery compound	2 kg	—
Linalool	AED 69.15	Customized	Perfumery compound	5 g	78-70-6

**Table 2:** Labeling chemicals present in the four samples of air fresheners.

*Air Freshener Sample 1*	
Aqua	Labeled
Isopropyl alcohol	Labeled
PEG 40 hydrogenated castor oil	Labeled
Parfum	Labeled
Lilial	Not labeled
Galaxolide	Not labeled

*Air Freshener Sample 2*	
Aqua	Labeled
Alcohol surfactant	Labeled
Fragrance	Labeled
Preservative	Labeled
Malodour counteracting (MOC) ingredient	Labeled
Antifoam	Labeled
Galaxolide	Not labeled
Benzenemethanol	Not labeled

*Air Freshener Sample 3*	
Butane	Labeled
SD alcohol 40-B	Labeled
Propane	Labeled
Fragrance	Labeled
Water	Labeled
Butylene glycol	Labeled
Camellia sinensis leaf extract	Labeled
Musk ketone	Not labeled
Benzenemethanol	Not labeled

*Air Freshener Sample 4*	
Butane	Labeled
Propane	Labeled
Isobutane	Labeled
Alcohol	Labeled
Parfum	Labeled
Lilial	Not labeled
Linalool	Not labeled
Butylated hydroxytoluene (BHT)	Not labeled

## Data Availability

The data (results of the measurements of toxic chemicals in the air fresheners) used to support the findings of this study are included within the article.
